# A reflection on the role of genetics in the concept of “epileptic encephalopathy”, as emerged from the most recent ILEA classification of epilepsy

**DOI:** 10.1186/s13052-019-0765-0

**Published:** 2020-01-06

**Authors:** Angelo Russo, Giuseppe Gobbi

**Affiliations:** 1 Department of Pediatric Neurology, IRCCS, Istituto delle Scienze Neurologiche di Bologna, UOC Neuropsichiatria Infantile, via Altura 3, Bologna, Italy; 2 Modena and Reggio Emilia University, Modena, Italy

**Keywords:** ILAE classification, Epileptic encephalopathy, Genetic epilepsy

## Abstract

The International League Against Epilepsy (ILAE) has been working to standardize the epilepsy classifications for over a hundred years.

The latest epilepsy classification has been recently carried out with a careful overview on several topics including the “epileptic encephalopathies” concept and several constructive discussions on this topic have taken place in the international community of epileptologists.

Here we wish to share our reflection on a statement of the ILAE commission on the “epileptic encephalopathy” concept, which in our opinion pays less attention to the “electroclinical syndromes” concept in favor of the new and very rapid genetic advances, thus generating confusion.

Our aim is both to preserve the role of electroclinical syndromes, while allowing for the association of the phenotype with specific gene mutations, and to underline the importance of bringing electroclinical syndromes back to the forefront of epileptology.

We believe the “match” is still open and for this reason we would like to share our considerations and to open a constructive debate on the “epileptic encephalopathy” concept.

## Background

In 1989 the concept of epilepsy syndromes was defined as “clusters of signs and symptoms customarily occurring together” [1]. This definition based on electroclinical features had the aim of facilitating clinical practice and research. Over time the “epilepsy syndromes” concept has evolved, incorporating issues related to comorbidities [2].

With the development of genetics, as well as next generation sequencing, many specific epileptic encephalopathies have been related to numerous genes considered causative. The risk linked to these genetic advances is to create a new form of specific epileptic encephalopathy for each gene that is found, when instead the found gene should indicate us exclusively the etiology of a given epileptic encephalopathy [2, 3].

The new ILAE classification stands as the mirror of modern epileptology, but our impression is that the modernity dictated by this very rapid progress of genetics is negatively influencing also some definitions contained in it, as for what concerns the concept of “epileptic encephalopathies” [3].

Here we would like to discuss the new proposal of ILAE on the “epileptic encephalopathies” concept and share our impressions to open a constructive debate on this topic.

## Overview on the “*Epileptic encephalopathy*” concept

Since 1909, the International League Against Epilepsy (ILAE) has made significant efforts to achieve better and internationally uniform classifications [1–10]. Recently, a new classification of epilepsies has been carried out with a careful overview on several topics, including the “epileptic encephalopathies” concept [11]. Over time this concept has opened several constructive discussions in the international community of epileptologists with important throwbacks, revisions and sometimes rejections [12–28]. We believe the “match” is still open and for this reason we would like to share our considerations and to open a constructive debate on this issue.

Gastaut (1973) in the *Dictionary of Epilepsy* defined Lennox-Gastaut syndrome as an “epileptic encephalopathy with diffuse slow-wave spikes, petit mal variant”. After 28 years (2001) the “epileptic encephalopathy” concept has been for the first time formally recognized by an ILAE Commission Report (J Engel) as “… a condition in which the epileptiform abnormalities themselves are believed to contribute to the progressive disturbance in cerebral function (new concept) …” , and then (2010) definitively taken over by Berg et al. (Report of the ILAE Commission on Classification and Terminology, 2005–2009) as the condition where “the epileptic activity itself contributes to severe cognitive and behavioral impairments above and beyond what might be expected from the underlying pathology alone (e.g., cortical malformation), and that these can worsen over time” [2, 6]. Cognitive and behavioral impairments can be seen along a spectrum of severity and they can occur at any age [2].

Furthermore, although various syndromes are often referred to as epileptic encephalopathies, the encephalopathic impact of seizures and epilepsy may potentially occur in association with any form of epilepsy [2] (up to now the recognized Epileptic Encephalopathies are: Early Myoclonic Encephalopathy, Early Infantile Epileptic Encephalopathy (Ohtahara Syndrome), Epilepsy of infancy with migrating focal seizures, West Syndrome, Dravet Syndrome, Epilepsy with Myoclonic Astatic Seizures, Lennox-Gastaut Syndrome, and Epilepsy with Continuous Spike-Waves during Slow Wave Sleep including Landau-Kleffner Syndrome and Atypical Benign Partial Epilepsy). In this regard, in the latest Classification of the Epilepsies (2017) the ILAE commission stated that “in an epileptic encephalopathy, the abundant epileptiform activity interferes with development resulting in cognitive slowing and often regression, and sometimes is associated with psychiatric and behavioural consequences” and underlined that the encephalopathic impact of the epileptiform activity could cause regression in patients with both normal development and pre-existing developmental delay, who then show developmental plateauing or regression.

This last point of view emphasizes the important assumption that improving the epileptic activity could reduce the developmental consequences.

Furthermore, the ILAE commission stated that “many of severe genetic disorders also have developmental consequences arising directly from the effect of the genetic mutation, in addition to the effect of the frequent epileptic activity on development”.

Starting from these considerations, the ILAE commission suggested three main ways in which the effect of the genetic mutation and the frequent epileptic activity could influence development: 1) “pre-existing developmental delay”, complicated by plateauing or regression; 2) “developmental slowing occurring on a background of normal development”, with the slowing emerging prior to the presence of frequent epileptic activity on the EEG, such as in Dravet Syndrome; 3) “permanent developmental impairment due to the severity of epilepsy”, even in cases where the latter starts relatively early, as it is seen in some patients with KCNQ2 or STXBP1 mutation.

On the basis of these last observations the ILAE suggested the inclusion of the word “developmental” in the description of encephalopathy leading to the definition of three main conditions: 1) *developmental encephalopathy where there is just developmental impairment without frequent epileptic activity associated with regression or further slowing of development*; 2) *epileptic encephalopathy where there is no pre-existing developmental delay and the genetic mutation is not thought to cause slowing in its own right; 3) developmental and epileptic encephalopathy where both factors play a role.*

Up to this point the reflection carried out by the ILAE commission is very clear and acceptable.

Our debate starts where the ILAE commission suggests analyzing the encephalopathic concept starting from a genetic point of view, following the recent and rapid genetic advances and stating that *“*where a genetic mutation of major effect is identified, the terms ‘developmental and epileptic encephalopathy’ may be subsumed by using the name of the underlying condition. For example, many of the well recognized developmental and epileptic encephalopathies can now be called by their gene name together with the word encephalopathy,, such as “STXBP1 encephalopathy*”* and furthermore stated that “when genes are associated with both severe and self-limited pharmacoresponsive epilepsies, such as KCNQ2 or SCN2A , then the term ‘encephalopathy’ can be used to denote the severe form*”* [11].

Therefore, following these statements we can talk about “SCN2A encephalopathy” only in the case of an early-onset infantile epileptic encephalopathy but not in the case of benign familial neonatal-infantile seizures or generalized epilepsy febrile seizures plus, and similarly we can talk about “KCNQ2 encephalopathy” only in the case of an early-onset epileptic encephalopathy with suppression burst or a late-onset epileptic encephalopathy with continuous spikes and waves during slow-wave sleep syndrome, but not in the case of benign familial neonatal seizures.

On the other hand, we know full well that some epileptic encephalopaties are associated with several genes. For instance, the Othahara syndrome could be due to mutations in the KCNQ2 and SCN2A genes and the Dravet Syndrome could be due to mutations in the SCN1A, SCN2A, SCN1B genes.

Therefore, our question for the ILAE Commission for Classification and Terminology is: when we talk about an SCN2A encephalopathy, are we referring to Dravet or Othahara syndrome? And when we talk about KCNQ2 encephalopathy, are we referring to the Othahara or ESES syndrome?

It seems clear that if the majority of genes show phenotypic heterogeneity and the majority of syndromes reveal genetic heterogeneity the interpretation of this heterogeneity and its significance need to be considered in the context of the electroclinical presentation, which should represent the only true starting point in the clinical practice of every epileptologist. In the current clinical practice, first we have to try to make the diagnosis and then we start to look for the etiology, including genetic abnormalities which can represent the etiology of a given electroclinical syndrome.

Following this reasoning we would suggest using the “*gene name* encephalopathy” nomenclature just as if we were encountering a genetic mutation in an unknown syndrome, such as with the CHD2 genes, while in a well-known syndrome, such as Dravet Syndrome or Othahara syndrome, we suggest using the “*syndrome name, gene name* encephalopathy” nomenclature, for instance “Dravet syndrome, SCN2A encephalopathy” or “Othahara syndrome, SCN2A encephalopathy”, and“Othahara syndrome, KCNQ2 encephalopathy” or “ESES syndrome, KCNQ2 encephalopathy”.

Furthermore, the whole scientific community recognizes the need to bring the role of clinical studies back to the forefront in order to obtain a better correlation between genotype and phenotype. Thus, it is expected that several entities that are currently indicated as “*gene name* encephalopathy” (e.g., CHD2 encephalopathy) will later change status and be addressed as “*syndrome name, gene name* encephalopathy”, when more will become known about their electroclinical features.

On the basis of these considerations, we suggest a clarification of the last point analyzed by the ILAE in the latest classification and we would like to open a constructive debate on the issue here discussed.

## Conclusion

Our reflection raises a doubt on the latest statement of the ILAE commission on the epileptic encephalopathy concept, which in our opinion reduces the importance of electroclinical syndromes in favor of the new and very rapid genetic advances, thus generating confusion.

To reduce this confusion, here we propose a possible simplification using the terminology “gene name encephalopathy” for still unknown syndromes caused by genetic mutations and “syndrome name, gene name encephalopathy” for well-known syndromes, such as Dravet syndrome.

The change in the terminology on which we are reflecting aims to preserve the role of electroclinical epileptic encephalopathy, a role still of extreme importance for the correct management of patients with epilepsy, but also a key role in the phenotypic and genetic correlation.

Further clarification on the “epileptic encephalopathy” concept is likely needed.

## Data Availability

Not applicable.
